# Marijuana, Opioid, and Alcohol Use Among Pregnant and Postpartum Individuals Living With HIV in the US

**DOI:** 10.1001/jamanetworkopen.2021.37162

**Published:** 2021-12-03

**Authors:** Lynn M. Yee, Deborah Kacanek, Chase Brightwell, Lisa B. Haddad, Jennifer Jao, Kathleen M. Powis, Tzy-Jyun Yao, Emily Barr, Carly Broadwell, Suzanne Siminski, George R. Seage, Ellen G. Chadwick

**Affiliations:** 1 Department of Obstetrics and Gynecology, Northwestern University Feinberg School of Medicine, Chicago, Illinois; 2 Center for Biostatistics in AIDS Research, Department of Biostatistics, Harvard T.H. Chan School of Public Health, Boston, Massachusetts; 3 Center for Biomedical Research, Population Council, New York, New York; 4 Department of Pediatrics, Northwestern University Feinberg School of Medicine, Chicago, Illinois; 5 Departments of Internal Medicine and Pediatrics, Massachusetts General Hospital, Boston; 6 Department of Immunology and Infectious Diseases, Harvard T.H. Chan School of Public Health, Boston, Massachusetts; 7 Department of Pediatric Infectious Diseases, University of Colorado School of Medicine, Aurora; 8 Frontier Science Foundation, Amherst, New York; 9 Department of Epidemiology, Harvard T.H. Chan School of Public Health, Boston, Massachusetts

## Abstract

**Importance:**

Amid the opioid epidemic and evolving legal and social changes with marijuana, little is known about substance use among pregnant and postpartum people living with HIV.

**Objectives:**

To evaluate trends in marijuana, alcohol, and opioid use during pregnancy and the first year postpartum among US people living with HIV and the differences in substance use based on marijuana legalization status.

**Design, Setting, and Participants:**

Data from the Surveillance Monitoring for Antiretroviral Toxicities (SMARTT) study of the Pediatric HIV/AIDS Cohort Study were analyzed. SMARTT-enrolled, pregnant people living with HIV at 22 US sites from January 1, 2007, to July 1, 2019, with self-reported substance use data available in pregnancy, 1 year postpartum, or both were assessed.

**Exposures:**

Calendar year and state marijuana legalization status.

**Main Outcomes and Measures:**

The prevalence of any use of each of the following substances was calculated by calendar year, separately for pregnancy and postpartum: marijuana, alcohol, opioid, and concomitant alcohol and marijuana. Log binomial models were fit using general estimating equations to evaluate the mean annual change, accounting for repeat pregnancies. The study also evaluated differences in substance use by state recreational or medical marijuana legalization status.

**Results:**

Substance use data were available for 2926 pregnancies from 2310 people living with HIV (mean [SD] age, 28.8 [6.1] years; 822 [28.1%] Hispanic, 1859 [63.5%] non-Hispanic Black, 185 [6.3%] White, 24 [0.8%] of more than 1 race, 24 [0.8%] of other race or ethnicity [individuals who identified as American Indian, Asian, or Native Hawaiian or other Pacific Islander], and 12 [0.4%] with unknown or unreported race or ethnicity). Between 2007 and 2019, marijuana use during pregnancy increased from 7.1% to 11.7%, whereas alcohol and opioid use in pregnancy were unchanged. Postpartum alcohol (44.4%), marijuana (13.6%), and concomitant alcohol and marijuana (10.0%) use were common; marijuana use increased from 10.2% to 23.7% from 2007 to 2019, whereas postpartum alcohol use was unchanged. The adjusted mean risk of marijuana use increased by 7% (95% CI, 3%-10%) per year during pregnancy and 11% (95% CI, 7%-16%) per year postpartum. Postpartum concomitant alcohol and marijuana use increased by 10% (95% CI, 5%-15%) per year. Differences in substance use were not associated with recreational legalization, but increased marijuana use was associated with medical marijuana legalization.

**Conclusions and Relevance:**

In this cohort study, opioid use among pregnant people living with HIV remained stable, whereas marijuana use during pregnancy and postpartum increased over time and in states with legalized medical marijuana. These patterns of increasing marijuana use among pregnant and postpartum people living with HIV suggest that enhanced clinical attention is warranted, given the potential maternal and child health implications of substance use.

## Key Points

**Question:**

What are the trends over time in marijuana, alcohol, and opioid use during pregnancy and the first year postpartum among US persons living with HIV?

**Findings:**

In this cohort study including 2310 pregnant and postpartum individuals living with HIV from the Surveillance Monitoring for Antiretroviral Toxicities (SMARTT) study, use of marijuana increased from 2007 to 2019 and was more frequent in locations with medical marijuana legalization. Prevalence of alcohol and opioid use did not change over that time.

**Meaning:**

These findings of increasing marijuana use among pregnant people living with HIV suggest that further clinical and research attention is warranted, given the potential implications for pregnancy and HIV-related health.

## Teaser

This cohort study evaluates trends over time in the prevalence of self-reported opioid, alcohol, and marijuana use during pregnancy and in the first year postpartum among people living with HIV and assesses the association of marijuana legalization status with substance use.

## Introduction

Marijuana use in the US has increased among pregnant and nonpregnant individuals of reproductive age.^1,2^ Legalization of marijuana for medical and recreational use, as well as increased social acceptance of marijuana use, may contribute to these trends.^3,4,5,6^ Marijuana use during pregnancy is common, is often perceived to be safe, and has even been recommended by dispensaries for alleviation of pregnancy symptoms.^3,4^ Despite professional guidelines that discourage the prescription or use of marijuana during preconception, pregnancy, and lactation, it remains commonly used.^3,7^ Although existing data suggest that marijuana use is not associated with fetal defects,^7,8,9^ use has been associated with alterations in birth weight and neurodevelopment and is associated with other psychosocial risk markers, including use of other substances, with implications for health.^7,9,10,11,12,13,14,15,16^

Concurrent with evolving marijuana use, the opioid crisis in the US, driven largely by use of prescription opioids, is a complex public health challenge that extends to pregnant and other reproductive-aged individuals.^17,18,19,20,21^ Maternal opioid use has increased steeply since 2000, with nearly 25% of pregnant people filling an opioid prescription.^20^ Opioid use and opioid use disorder have substantial implications for perinatal and long-term health.^20^ Moreover, the immediate postpartum period, when opioids may be prescribed to manage pain, introduces the risk of persistent opioid use.^22,23,24,25,26,27,28^

Examining substance use among pregnant and postpartum persons living with HIV is particularly important because these individuals experience comorbid medical and psychosocial conditions that enhance the risk of adverse perinatal outcomes. Although trends in substance use have been described among pregnant persons without HIV and nonpregnant persons living with HIV in the US,^16,29^ similar evaluations specific to pregnant and postpartum people living with HIV have not been performed.^2^ Thus, the objectives of this study were to (1) evaluate trends over time in the prevalence of self-reported opioid, alcohol, and marijuana use during pregnancy and in the first year postpartum among people living with HIV in the US and (2) assess the association of state medical or recreational marijuana legalization status with substance use. This analysis also examined alcohol because there have been no changes in medical guidelines regarding alcohol since the long-standing recommendation to abstain from all alcohol use during pregnancy.^30^ Thus, changes in alcohol use are a useful comparator against which to assess changes in polysubstance use over time.

## Methods

Data from the Surveillance Monitoring for Antiretroviral Toxicities Study (SMARTT) study, a multicenter, prospective cohort study of the National Institutes of Health–funded Pediatric HIV/AIDS Cohort Study, were used to evaluate substance use among pregnant and postpartum people living with HIV. SMARTT has enrolled pregnant and postpartum people living with HIV at 22 US clinical sites (eAppendix in the Supplement) since 2007. SMARTT aims to study the health of children with perinatal exposure to HIV and antiretroviral drugs but who remain uninfected, as well as the health of their caregivers. The SMARTT Dynamic cohort enrolls mother-newborn pairs during pregnancy or within 72 hours of delivery with collection of sociobehavioral, medical, and biological data at enrollment and annually. Each participant provided written informed consent for SMARTT participation. Data were deidentified for this analysis. The institutional review board at the Harvard T.H. Chan School of Public Health and each site approved the study. The study followed the Strengthening the Reporting of Observational Studies in Epidemiology (STROBE) reporting guideline.

Eligible for this analysis were SMARTT Dynamic cohort people living with HIV enrolled from January 1, 2007, to July 1, 2019, with data available during pregnancy, postpartum, or in both periods. Outcomes of interest included the prevalence of opioid, marijuana, alcohol, and concomitant alcohol and marijuana use. All substance use was self-reported via structured interviews conducted in person by trained personnel. Interviews, administered between the delivery date and up to 7 days after the participant gave birth, assessed substance use during pregnancy (ever use), including trimester of use and frequency of use for each substance; these interviews did not use a validated screening tool. At 1 year postpartum, the Client Diagnostic Questionnaire, a mental health screening tool designed for people living with HIV,^31,32^ assessed the frequency of substance use in the previous 6 months for each substance the individual reported having ever used.^31^

Opioid use during pregnancy was defined as at least 1 affirmative response regarding use of narcotic pain medications, heroin, opium, or methadone during pregnancy. Alcohol use was defined as affirmative responses regarding any alcohol use during pregnancy or postpartum. Marijuana use was defined as affirmative responses regarding marijuana use at any time during pregnancy or postpartum. For opioids and marijuana, prescribed vs nonprescribed substance use was not available in these data. Because of the high reported rate of concomitant substance use in the general population, particularly alcohol and marijuana,^13,33,34^ we also evaluated concomitant use of both alcohol and marijuana (a subset of those reporting alcohol or marijuana use, respectively), although concomitant substance use does not necessarily indicate simultaneous use. To evaluate intensity, alcohol use was classified as hazardous if the individual had 28 or more drinks per month or at least 4 drinks consumed on a single occasion.^35^ Frequent marijuana use was defined as 3 or more times per week.

For the evaluation of substance use trends over time, the exposure of interest was delivery year. For the evaluation of legalization, the exposures of interest were recreational and medical marijuana legalization status (evaluated separately) based on state of residence at the time of delivery and year of delivery (eFigure 1 in the Supplement). Only marijuana and alcohol use were evaluated for this objective, given the focus on marijuana legalization. Dates of marijuana legalization were obtained from the Marijuana Policy Project^36^ and confirmed via search of individual state legislation data. For this objective, pregnancies were classified into 3 categories comprising state recreational or medical legalization status: never legal, before legalization (ie, occurring before legalization in states that later legalized marijuana use), and after legalization (ie, occurring after legalization in states that had already legalized marijuana use). In states where legalization occurred during the study period, pregnancies for which the date at last menstrual period was the same or later than the date of marijuana legalization were classified as after legalization. Pregnancies with last menstrual period dates that occurred before the legalization date were classified as before legalization. Pregnancies that occurred in states where medical or recreational marijuana had never been legalized were categorized as never legal. Similarly, postpartum periods were separated into 3 categories of state recreational or medical legalization status, using the delivery date as referent date.

Maternal demographic and clinical characteristics were described overall and according to availability of substance use data (pregnancy only, postpartum only, or both periods). Pregnancy and postpartum analyses were performed separately for all outcomes, with the exception of opioids, for which postpartum use data were not available in SMARTT (with the exception of heroin). The prevalence and 95% CIs of any use for each substance in pregnancy (all drugs) and in the first year postpartum (alcohol and marijuana only) were estimated by year of delivery, using 1-year intervals. To evaluate the mean annual change in prevalence of substance use, unadjusted and adjusted log binomial regression models using generalized estimating equations were fit to estimate the relative risk (RR) of substance use per additional calendar year, accounting for additional pregnancies within persons. Multivariable models adjusted for maternal age at conception and self-reported race and ethnicity, chosen a priori based on clinical importance. Race and ethnicity were included in multivariable models because they represent social determinants of health that may serve as potential confounders. Additional descriptive analyses summarized trimester of use and, for postpartum data, intensity of substance use. For the analysis of differences in use by legalization status, log binomial regression models using generalized estimating equations were fit to estimate the association of legalization status with each outcome (marijuana, alcohol, as well as concomitant alcohol and marijuana use) in each period. Multivariable models included potential confounders chosen a priori, including maternal age, self-reported race and ethnicity, and household income. Analysis was performed in SAS statistical software, version 9.2 (SAS Institute Inc).

## Results

Of 3085 SMARTT Dynamic pregnancies delivered as of July 1, 2019, a total of 2926 pregnancies among 2310 people living with HIV (mean [SD] age, 28.8 [6.1] years; 822 [28.1%] Hispanic, 1859 [63.5%] non-Hispanic Black, 185 [6.3%] White, 24 [0.8%] of more than 1 race, 24 [0.8%] of other race or ethnicity [individuals who identified as American Indian, Asian, or Native Hawaiian or other Pacific Islander], and 12 [0.4%] with unknown or unreported race or ethnicity) were eligible for inclusion in this analysis (eFigure 2 in the Supplement). Of 2926 pregnancies, 21 had substance use data postpartum but not in pregnancy, 1131 had substance use data in pregnancy but not postpartum, and 1774 had both pregnancy and postpartum substance use data. Household income was reported as $20 000 or less per year by 62.7% participants, with 68.9% reporting at least a high school education. A total of 1341 pregnancies occurred before legalization of medical marijuana, whereas 919 occurred after medical marijuana legalization, and 666 were in locations where medical marijuana was never legal (Table 1). Regarding recreational legalization, 2397 pregnancies occurred in places where recreational marijuana was never legal, but 403 occurred before recreational marijuana legalization and 126 occurred after.

**Table 1.** Demographic and Clinical Characteristics of Pregnant and Postpartum Persons Living With HIV by Medical Marijuana Legalization Status During Pregnancy From 2007 to 2019^a^

Characteristic	Total (N = 2926)	Never legal medical marijuana use (n = 666)	Before medical marijuana legalization (n = 1341)	After medical marijuana legalization (n = 919)
Age at conception, mean (SD), y	28.8 (6.1)	28.1 (5.6)	28.3 (6.3)	30.1 (5.9)
Race and ethnicity				
Hispanic (regardless of race)	822 (28.1)	29 (4.4)	410 (30.6)	383 (41.7)
Non-Hispanic Black	1859 (63.5)	580 (87.1)	846 (63.1)	433 (47.1)
Non-Hispanic White	185 (6.3)	53 (8.0)	54 (4.0)	78 (8.5)
>1 Race	24 (0.8)	4 (0.6)	13 (1.0)	7 (0.8)
Other^b^	24 (0.8)	0	8 (0.6)	16 (1.7)
Unknown or not reported	12 (0.4)	0	10 (0.7)	2 (0.2)
Annual household income, $				
<10 000	1210 (41.4)	308 (46.2)	562 (41.9)	340 (37.0)
10 001-20 000	623 (21.3)	121 (18.2)	291 (21.7)	211 (23.0)
20 001-30 000	334 (11.4)	89 (13.4)	126 (9.4)	119 (12.9)
≥30 001	395 (13.5)	98 (14.7)	138 (10.3)	159 (17.3)
Unknown	364 (12.4)	50 (7.5)	224 (16.7)	90 (9.8)
Highest educational level of high school or above	2001 (68.9)	505 (76.1)	891 (66.9)	605 (66.7)
Vaginal delivery	1235 (42.7)	311 (47.8)	462 (34.6)	462 (50.8)
State				
New York	504 (17.2)	0	376 (28.0)	128 (13.9)
Alabama	155 (5.3)	155 (23.3)	0	0
California	364 (12.4)	0	0	364 (39.6)
Puerto Rico	211 (7.2)	0	179 (13.3)	32 (3.5)
Tennessee	274 (9.4)	274 (41.1)	0	0
Colorado	165 (5.6)	0	0	165 (18.0)
New Jersey	114 (3.9)	0	47 (3.5)	67 (7.3)
Florida	616 (21.1)	0	555 (41.4)	61 (6.6)
Illinois	252 (8.6)	0	150 (11.2)	102 (11.1)
Pennsylvania	20 (0.7)	0	20 (1.5)	0
Texas	144 (4.9)	144 (21.6)	0	0
Louisiana	93 (3.2)	92 (14.0)	0	0
Maryland	14 (0.5)	0	14 (1.0)	0

^a^ Data are presented as number (percentage) of participants unless otherwise indicated.

^b^ Includes individuals who identified as American Indian (n = 7), Asian (n = 16), or Native Hawaiian or other Pacific Islander (n = 1).

The prevalence of marijuana use during pregnancy was 9.3% overall, increasing from 7.1% in 2007-2008 to 11.7% in 2018-2019 and peaking at 13.3% in 2013 (eFigure 3 in the Supplement). The overall prevalence of opioid use (5.8% in 2007-2008 to 3.9% in 2018-2019, peaking at 8.7% in 2012), alcohol use (8.6% in 2007-2008 to 8.2% in 2018-2019, peaking at 11.8% in 2013), and concomitant alcohol and marijuana use (1.8% in 2007-2008 to 2.8% in 2018-2019, peaking at 5.2% in 2014) during pregnancy remained stable during the study period (eTable in the Supplement). Postpartum substance use was more common, with a mean prevalence of 44.4% for alcohol, 13.6% for marijuana, and 10.0% for concomitant alcohol and marijuana. As with pregnancy, postpartum data demonstrated increases in marijuana use over time, from 10.2% in 2007-2008 to 23.7% in 2018-2019 (Figure). Postpartum alcohol use increased from 2007-2008 (36.2%) to 2012 (53.8%) but returned to baseline levels by 2018-2019 (42.1%). Concomitant postpartum alcohol and marijuana use increased from 6.7% in 2007-2008 to 15.8% in 2018-2019.

**Figure.**
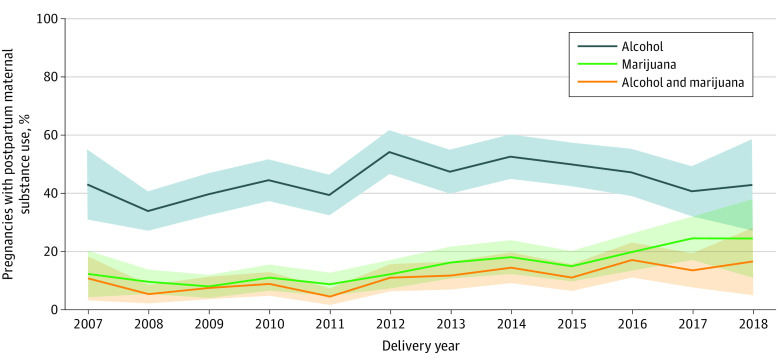
Prevalence of Substance Use in the First Year Postpartum Among Persons Living With HIV by Delivery Year From 2007 to 2019 Shaded areas indicate 95% CIs.

Adjusted analyses of substance use demonstrated mean annual 7% increase in marijuana use (adjusted RR, 1.07; 95% CI, 1.03-1.10) during pregnancy, although no significant mean annual changes were identified for other substances (Table 2). In the postpartum period, an 11% mean annual increase was observed in marijuana use (adjusted RR, 1.11; 95% CI, 1.07-1.16) and a 10% mean annual increase in concomitant alcohol and marijuana (adjusted RR, 1.10; 95% CI, 1.05-1.15), but no increase was seen in alcohol use only (Table 2).

**Table 2.** Unadjusted and Adjusted Average Annual RRs of Substance Use Among Pregnant and Postpartum Persons Living With HIV From 2007 to 2019

Substance	RR (95% CI) per calendar year	
	Unadjusted	Adjusted^a^
**Pregnancy**		
Alcohol	0.99 (0.96-1.02)	0.99 (0.95-1.02)
Marijuana	1.06 (1.02-1.10)	1.07 (1.03-1.10)
Alcohol and marijuana	1.02 (0.97-1.09)	1.02 (0.97-1.09)
Opioids	0.97 (0.93-1.02)	0.97 (0.93-1.02)
**Postpartum**		
Alcohol	1.02 (1.00-1.04)	1.02 (1.00-1.04)
Marijuana	1.11 (1.06-1.15)	1.11 (1.07-1.16)
Alcohol and marijuana	1.10 (1.05-1.15)	1.10 (1.05-1.15)

Abbreviation: RR, relative risk.

^a^ Adjusted for maternal self-reported race, ethnicity, and age, accounting for additional pregnancies.

Alcohol consumption during pregnancy was most frequent in the first trimester (8.5% in the first trimester, 2.5% in the second trimester, and 1.9% in the third trimester). Marijuana consumption during pregnancy was 8.3% in the first trimester, 4.3% in the second trimester, and 3.2% in the third trimester. In contrast, opioid use did not vary significantly by trimester (2.5% in the first trimester, 2.9% in the second trimester, and 2.8% in the third trimester). In the postpartum period, the proportion of individuals reporting hazardous drinking increased from 6.2% in 2007-2008 to 11.6% in 2016 and returned to 7.9% in 2018-2019. Frequent marijuana use in the postpartum period differed by time; in 2008, 3.2% of individuals reported using marijuana 3 or more times per week, which increased to 9.1% in 2015 and 18.4% in 2018.

No differences in substance use during pregnancy were identified by recreational marijuana legalization (Table 3). However, when evaluating by medical marijuana legalization status, marijuana use was more prevalent among pregnancies occurring after vs before legalization (adjusted RR, 1.36; 95% CI, 1.02-1.80). Conversely, alcohol use during pregnancy was less frequent among persons experiencing pregnancies after medical marijuana legalization (adjusted RR, 0.68; 95% CI, 0.52-0.90) or in never-legal states (adjusted RR, 0.60; 95% CI, 0.43-0.84) compared with those occurring in states before medical marijuana legalization. Similarly, the adjusted RR of concomitant alcohol and marijuana use was lower among pregnancies in areas where medical marijuana was never legal than among pregnancies that occurred before legalization (adjusted RR, 0.37; 95% CI, 0.16-0.83).

**Table 3.** Substance Use During Pregnancy by State Marijuana Legalization Status Among Pregnant Persons Living With HIV From 2007 to 2019

State marijuana legalization status	Reported use of substance, No./total No. (%)	RR (95% CI)	
		Unadjusted model	Adjusted model^a^
**Recreational marijuana**			
Alcohol			
Before legalization	31/400 (7.8)	1 [Reference]	1 [Reference]
After legalization	14/125 (11.2)	1.44 (0.78-2.66)	1.37 (0.74-2.55)
Never legal^b^	227/2378 (9.5)	1.23 (0.86-1.78)	1.35 (0.93-1.96)
Marijuana			
Before legalization	39/400 (9.8)	1 [Reference]	1 [Reference]
After legalization	17/125 (13.6)	1.39 (0.78-2.48)	1.30 (0.74-2.29)
Never legal^b^	214/2378 (9.0)	0.92 (0.66-1.29)	0.72 (0.51-1.02)
Alcohol and marijuana			
Before legalization	13/400 (3.3)	1 [Reference]	1 [Reference]
After legalization	6/125 (4.8)	1.48 (0.57-3.79)	1.35 (0.51-3.56)
Never legal^b^	61/2378 (2.6)	0.79 (0.43-1.44)	0.72 (0.39-1.34)
**Medical marijuana**			
Alcohol			
Before legalization^c^	151/1326 (11.4)	1 [Reference]	1 [Reference]
After legalization	76/915 (8.4)	0.73 (0.55-0.96)	0.68 (0.52-0.90)
Never legal	45/662 (6.8)	0.60 (0.43-0.83)	0.60 (0.43-0.84)
Marijuana			
Before legalization^c^	118/1326 (8.9)	1 [Reference]	1 [Reference]
After legalization	94/916 (10.3)	1.15 (0.87-1.53)	1.36 (1.02-1.80)
Never legal	58/662 (8.8)	0.98 (0.71-1.37)	0.84 (0.60-1.19)
Alcohol and marijuana			
Before legalization^c^	43/1326 (3.2)	1 [Reference]	1 [Reference]
After legalization	28/915 (3.1)	0.94 (0.59-1.54)	0.97 (0.59-1.60)
Never legal	9/662 (1.4)	0.42 (0.19-0.92)	0.37 (0.16-0.83)

Abbreviation: RR, relative risk.

^a^ Models adjusted for maternal race, ethnicity, age, and income, accounting for additional pregnancies.

^b^ Data on 2 pregnancies were missing in states where recreational marijuana was never legal.

^c^ Data on 2 pregnancies were missing in states where medical marijuana had before-legalization status.

In the postpartum period, no differences in substance use by recreational marijuana legalization status were noted (Table 4). However, the adjusted RRs of marijuana and concomitant alcohol and marijuana use were both higher among postpartum people living with HIV who gave birth in states after vs before medical marijuana legalization (adjusted RR, 1.84; 95% CI, 1.40-2.42 for marijuana; adjusted RR, 1.75; 95% CI, 1.25-2.45 for alcohol and marijuana). No differences in postpartum use were noted when comparing those in never-legal states with those giving birth in states before legalization.

**Table 4.** Substance Use During the First Postpartum Year by State Marijuana Legalization Status Among Postpartum Persons Living With HIV From 2007 to 2019

State marijuana legalization status	Reported use of substance, No./Total No. (%)	RR (95% CI)	
		Unadjusted model	Adjusted model^a^
**Recreational marijuana^b^**			
Alcohol			
Before legalization	93/226 (41.2)	1 [Reference]	1 [Reference]
After legalization	35/89 (39.3)	0.96 (0.71-1.29)	0.89 (0.67-1.19)
Never legal	665/1473 (45.1)	1.10 (0.91-1.32)	1.02 (0.85-1.23)
Marijuana			
Before legalization	28/228 (12.3)	1 [Reference]	1 [Reference]
After legalization	13/89 (14.6)	1.19 (0.63-2.24)	1.00 (0.54-1.85)
Never legal	203/1478 (13.7)	1.12 (0.74-1.69)	0.76 (0.50-1.16)
Alcohol and marijuana			
Before legalization	23/226 (10.2)	1 [Reference]	1 [Reference]
After legalization	11/89 (12.4)	1.21 (0.60-2.47)	0.99 (0.49-1.99)
Never legal	144/1473 (9.8)	0.96 (0.60-1.55)	0.68 (0.41-1.12)
**Medical marijuana^c^**			
Alcohol			
Before legalization	359/835 (43.0)	1 [Reference]	1 [Reference]
After legalization	237/529 (44.8)	1.04 (0.91-1.19)	1.05 (0.92-1.20)
Never legal	197/424 (46.5)	1.08 (0.94-1.24)	0.99 (0.86-1.15)
Marijuana			
Before legalization	93/836 (11.1)	1 [Reference]	1 [Reference]
After legalization	89/532 (16.7)	1.50 (1.13-2.00)	1.84 (1.40-2.42)
Never legal	62/427 (14.5)	1.30 (0.95-1.80)	1.06 (0.77-1.47)
Alcohol and marijuana			
Before legalization	72/835 (8.6)	1 [Reference]	1 [Reference]
After legalization	67/529 (12.7)	1.47 (1.05-2.06)	1.75 (1.25-2.45)
Never legal	39/424 (9.2)	1.07 (0.71-1.59)	0.87 (0.58-1.30)

Abbreviation: RR, relative risk.

^a^ Models adjusted for maternal race, ethnicity, age, and income, accounting for additional pregnancies.

^b^ Data from 2 pregnancies were missing from the states with before-legalization status for alcohol data and 5 pregnancies with marijuana data from states with never-legal recreational marijuana.

^c^ Data were missing from 7 pregnancies for alcohol: 3 from states with never-legal status, 1 from a state with before-legalization status, and 3 from states with after-legalization status.

## Discussion

In this cohort study, we hypothesized that marijuana and opioid use in this population may mirror national trends with increasing use. However, opioid use during pregnancy in this population was uncommon, with no appreciable changes during the 12-year study period. In contrast, marijuana use increased throughout the study period for pregnant and postpartum people living with HIV, along with concomitant alcohol and marijuana use among postpartum people living with HIV. Marijuana use was higher among persons living in states and during periods with medical marijuana legalization. Understanding how the opioid crisis and evolving legal policies on marijuana are associated with substance use among pregnant and postpartum people living with HIV has important public health implications for pregnancy- and HIV-related health.

### Results in Context

In an earlier analysis of SMARTT data, Rough et al^37^ demonstrated that overall substance use rates decreased sharply from 1990 to 2012 in pregnant people living with HIV, although substance use was defined more broadly (tobacco, alcohol, marijuana, cocaine, heroin, and injected drugs). In that analysis, the prevalence of substance use during pregnancy among people living with HIV was low for most substances and comparable to the general US population.^37^ However, these earlier data predated the current opioid epidemic among pregnant people and the most recent sociopolitical changes in marijuana use.^7,15,20,38,39^ As in the study by Rough et al,^37^ self-reported opioid use was low in this population. Although one reason may be underreporting, another reason may be that SMARTT sites do not completely overlap with the regions most affected by the opioid epidemic or that individuals with greater opioid use may not enroll in studies such as SMARTT. In contrast, increased reported use of marijuana among people living with HIV, particularly in the postpartum period, represents a novel finding. Reasons for this trend are myriad; marijuana use may reflect efforts to alleviate adverse effects of antiretroviral therapy or an effort to self-medicate for early pregnancy or mental health symptoms.^40^ Further research must identify the reasons people use marijuana in pregnancy and postpartum to inform interventions that improve birth outcomes and contribute to sustained engagement in HIV care.

Comparison of these findings to other contemporary populations of people living with HIV suggest that differences in substance use likely exist based on cohort characteristics. For example, data from the Women’s Interagency HIV Study^41^ reflect an older population of people living with HIV (mean age, 48 years); in contrast to our findings, the previous study^41^ identified a 22.2% prevalence of marijuana use and a 2.0% prevalence of opioid use. The Women’s Interagency HIV Study reported approximately 1 in 5 people living with HIV met the criteria for hazardous alcohol consumption, a much greater prevalence than in this analysis.^42^ These differences may be attributable to different behaviors in pregnant vs nonpregnant cohorts. Understanding such differences may be particularly important among pregnant people living with HIV, for whom substance use during pregnancy may carry even greater maternal-fetal implications.^43^ In addition, the relatively high prevalence of concomitant alcohol and marijuana use mirrors data from pregnant individuals without HIV.^2,13^

### Clinical and Research Implications

Despite some mixed results, the findings of this cohort study are consistent with accumulating evidence in nonpregnant populations that suggest increased marijuana use with medical marijuana legalization.^2,44^ Few studies have examined substance use during pregnancy and the postpartum period by legalization status. Although we did not identify differences by recreational marijuana legalization, the sample size was small, and findings may evolve as recreational legalization expands. For example, in a small California study,^45^ marijuana use in pregnancy increased after recreational legalization, and a larger study^46^ demonstrated increased use in the preconception, prenatal, and postpartum periods in states with recreational marijuana legalization compared with states without. In addition, substance use treatment facility admissions during pregnancy increased after medical marijuana law implementation.^47^ Given widespread medical marijuana legalization and the evolving climate of recreational marijuana legalization, counseling and messaging regarding the potential harms of marijuana use during and after pregnancy must remain a public health priority. In addition, the potential association of marijuana use with adherence to antiretroviral therapy must be considered; although marijuana may be favorably associated with adherence if it alleviates nausea, for example, the potential deleterious associations of substance use with regard to perinatal HIV transmission represent important aspects of counseling.

These findings highlight critical lessons about optimizing care for pregnant and postpartum people living with HIV. First, substance use was most common in the first trimester and in the postpartum period. These findings are consistent with other data that indicate that substance use prevalence decreases with advancing gestation, potentially because of alleviation of nausea or awareness of pregnancy.^13^ Our data highlight the importance of integrating substance use counseling with family planning care for people living with HIV, for whom timing pregnancy is essential because of the importance of optimization of antiretroviral therapy before conception as well as optimization of other determinants of perinatal health. Second, the high prevalence and increasing frequency of postpartum marijuana use provide a call to action for practitioners of interconception care to counsel patients about reducing substance use for maternal and family health, even after the heightened risk period of pregnancy is over.

### Limitations

This study has some limitations. Substance use data were collected by self-report, which is subject to social desirability bias. Some participants may have altered their responses over concern that reporting of substance use in pregnancy may have prompted newborn custody challenges. This issue may differentially influence reporting based on state legalization status; less frequent substance use in states with never-legal marijuana status may reflect lower use, perhaps because of the legal consequences of use, or may reflect underreporting. However, a prior study^48^ in SMARTT found self-reported substance use in pregnancy to be highly correlated with levels of marijuana (sensitivity, 80%; specificity, 98%; Cohen κ = 0.61) as well as tobacco and cocaine in offspring meconium. The SMARTT study lacks data on postpartum opioid use or indications for substance use, precluding analysis of prescribed marijuana or opioids. We are additionally unable to confirm that we have identified all forms of marijuana used. Furthermore, these data represent people living with HIV in perinatal care who enrolled in a longitudinal study of children born exposed to HIV but who remain uninfected, which may limit generalizability; these individuals resided in states that were less affected by the opioid epidemic. Future studies should address other populations of people living with HIV, including those living in states with a higher prevalence of opioid use. Finally, the analysis of recreational marijuana legalization is limited by small sample sizes because only 2 SMARTT sites were in states with legal recreational marijuana use during the study period. Despite these limitations, the large, diverse sample of pregnant and postpartum people living with HIV and the prospective collection of substance use data permitting comparisons by marijuana legalization status are major study strengths.^15^

## Conclusions

Although opioid use among pregnant people living with HIV was stable, marijuana use in this cohort increased, and medical marijuana legalization may be associated with increased marijuana use in this population. These patterns of increasing marijuana use among pregnant and postpartum people living with HIV warrant enhanced clinical attention given the potential maternal and child health implications of substance use. These results suggest that future work should investigate postpartum opioid use, the longitudinal patterns of use from pregnancy to postpartum, and the association of use with expanded recreational marijuana legalization.

## Notes

Supplement. **eAppendix.** Supplemental Information **eFigure 1.** Marijuana Legalization Status for Surveillance Monitoring for Antiretroviral Therapy Toxicities (SMARTT) Study Sites as of 2019 **eFigure 2.** Study Population Derivation **eFigure 3.** Prevalence and 95% Confidence Intervals of Substance Use During Pregnancy Among Persons Living With HIV by Delivery Year From 2007 to 2019 **eTable.** Substance Use During Pregnancy and Postpartum by Year of Delivery Among Pregnant People Living With HIV From 2007 to 2019
