# Supplementary material for: Marijuana, Opioid, and Alcohol Use Among Pregnant and Postpartum Individuals Living With HIV in the US
Source: JAMA Netw Open. 2021 Dec 3;4(12):e2137162. doi: 10.1001/jamanetworkopen.2021.37162 (PMC8642784; doi:10.1001/jamanetworkopen.2021.37162)
Supplement: Supplement. — eAppendix. Supplemental Information eFigure 1. Marijuana Legalization Status for Surveillance Monitoring for Antiretroviral Therapy Toxicities (SMARTT) Study Sites as of 2019 eFigure 2. Study Population Derivation eFigure 3. Prevalence and 95% Confidence Intervals of Substance Use During Pregnancy Among Persons Living With HIV by Delivery Year From 2007 to 2019 eTable. Substance Use During Pregnancy and Postpartum by Year of Delivery Among Pregnant People Living With HIV From 2007 to 2019 [file jamanetwopen-e2137162-s001.pdf]

Supplementary Online Content

Yee LM, Kacanek D, Brightwell C, et al. Marijuana, opioid, and alcohol use among pregnant and postpartum individuals living with HIV in the US. *JAMA Netw Open*. 2021;4(12):e2137162. doi:10.1001/jamanetworkopen.2021.37162

eAppendix. Supplemental Information

eFigure 1. Marijuana Legalization Status for Surveillance Monitoring for Antiretroviral Therapy Toxicities (SMARTT) Study Sites as of 2019

eFigure 2. Study Population Derivation

eFigure 3. Prevalence and 95% Confidence Intervals of Substance Use During Pregnancy Among Persons Living With HIV by Delivery Year From 2007 to 2019

eTable. Substance Use During Pregnancy and Postpartum by Year of Delivery Among Pregnant People Living With HIV From 2007 to 2019

This supplementary material has been provided by the authors to give readers additional information about their work.

© 2021 Yee LM et al. *JAMA Network Open.*

eAppendix. Supplemental Information

We thank the women for their participation in PHACS, and the individuals and institutions involved in the conduct of PHACS. The study was supported by the Eunice Kennedy Shriver National Institute of Child Health & Human Development (NICHD), Office of The Director, National Institutes of Health (OD), National Institute of Dental & Craniofacial Research (NIDCR), National Institute of Allergy And Infectious Diseases (NIAID), National Institute of Neurological Disorders And Stroke (NINDS), National Institute on Deafness And Other Communication Disorders (NIDCD), National Institute of Mental Health (NIMH), National Institute on Drug Abuse (NIDA), National Cancer Institute (NCI), and National Institute on Alcohol Abuse And Alcoholism (NIAAA) through cooperative agreements with the Harvard T.H. Chan School of Public Health (HD052102) (Principal Investigator: George R Seage III; Program Director: Liz Salomon) and the Tulane University School of Medicine (HD052104) (Principal Investigator: Russell Van Dyke; Co-Principal Investigator: Ellen Chadwick; Project Director: Patrick Davis), and through P01HD103133 with Harvard T.H. Chan School of Public Health for the Pediatric HIV/AIDS Cohort Study (PHACS) 2020 (Multiple Principal Investigators: George R Seage III, Ellen Chadwick; Program Director: Liz Salomon). Data management services were provided by Frontier Science (Data Management Center Director: Suzanne Siminski), and regulatory services and logistical support were provided by Westat, Inc (Project Directors: Julie Davidson, Tracy Wolbach).

The following institutions, clinical site investigators and staff participated in conducting PHACS SMARTT in 2020, in alphabetical order: **Ann & Robert H. Lurie Children's Hospital of Chicago**: Ellen Chadwick, Margaret Ann Sanders, Kathleen Malee; **Baylor College of Medicine**: Mary Paul, Ruth Eser-Jose, Chivon McMullen-Jackson, Lynnette Harris; **BronxCare Health System:** Murli Purswani, Mahoobullah Mirza Baig, Alma Villegas, Marvin Alvarado; **Children's Diagnostic & Treatment Center**: Lisa-Gaye Robinson, Jawara Dia Cooley, James Blood, Patricia Garvie; **New York University School of Medicine**: William

© 2021 Yee LM et al. *JAMA Network Open.*

Borkowsky, Nagamah Sandra Deygoo, Jennifer Lewis; **Rutgers - New Jersey Medical School:** Arry Dieudonne, Linda Bettica, Juliette Johnson, Karen Surowiec; **St. Jude Children's Research Hospital**: Katherine Knapp, Jamie Russell-Bell, Megan Wilkins, Stephanie Love; **San Juan Hospital Research Unit/Department of Pediatrics, San Juan Puerto Rico:** Nicolas Rosario, Lourdes Angeli-Nieves, Vivian Olivera; **SUNY Downstate Medical Center**: Stephan Kohlhoff, Ava Dennie, Jean Kaye, Jenny Wallier; **Tulane University School of Medicine**: Karen Craig, Russell Van Dyke, Patricia Sirois; **University of Alabama, Birmingham**: Cecelia Hutto, Paige Hickman, Julie Huldtquist, Dan Marullo; **University of California, San Diego**: Stephen A. Spector, Veronica Figueroa, Megan Loughran, Sharon Nichols; **University of Colorado, Denver:** Elizabeth McFarland, Christine Kwon, Carrie Glenny, Jennifer Englund; **University of Florida, Center for HIV/AIDS Research, Education and Service:** Mobeen Rathore, Saniyyah Mahmoudi, Sarah El-Hassan, Jamilah Tejan; **University of Illinois, Chicago:** Karen Hayani, Lourdes Richardson, Renee Smith, Alina Miller; **University of Miami:** Gwendolyn Scott, Gustavo Gil Garcia, Gabriel Fernandez, Anai Cuadra; **Keck Medicine of the University of Southern California:** Toni Frederick, Mariam Davtyan, Guadalupe Morales-Avendano; **University of Puerto Rico School of Medicine, Medical Science Campus**: Zoe M. Rodriguez, Lizmarie Torres, Nydia Scalley

Note: The conclusions and opinions expressed in this article are those of the authors and do not necessarily reflect those of the National Institutes of Health or U.S. Department of Health and Human Services.

eFigure 1. Marijuana Legalization Status for Surveillance Monitoring For Antiretroviral Therapy Toxicities (SMARTT) Study Sites as of 2019

© 2021 Yee LM et al. *JAMA Network Open.*

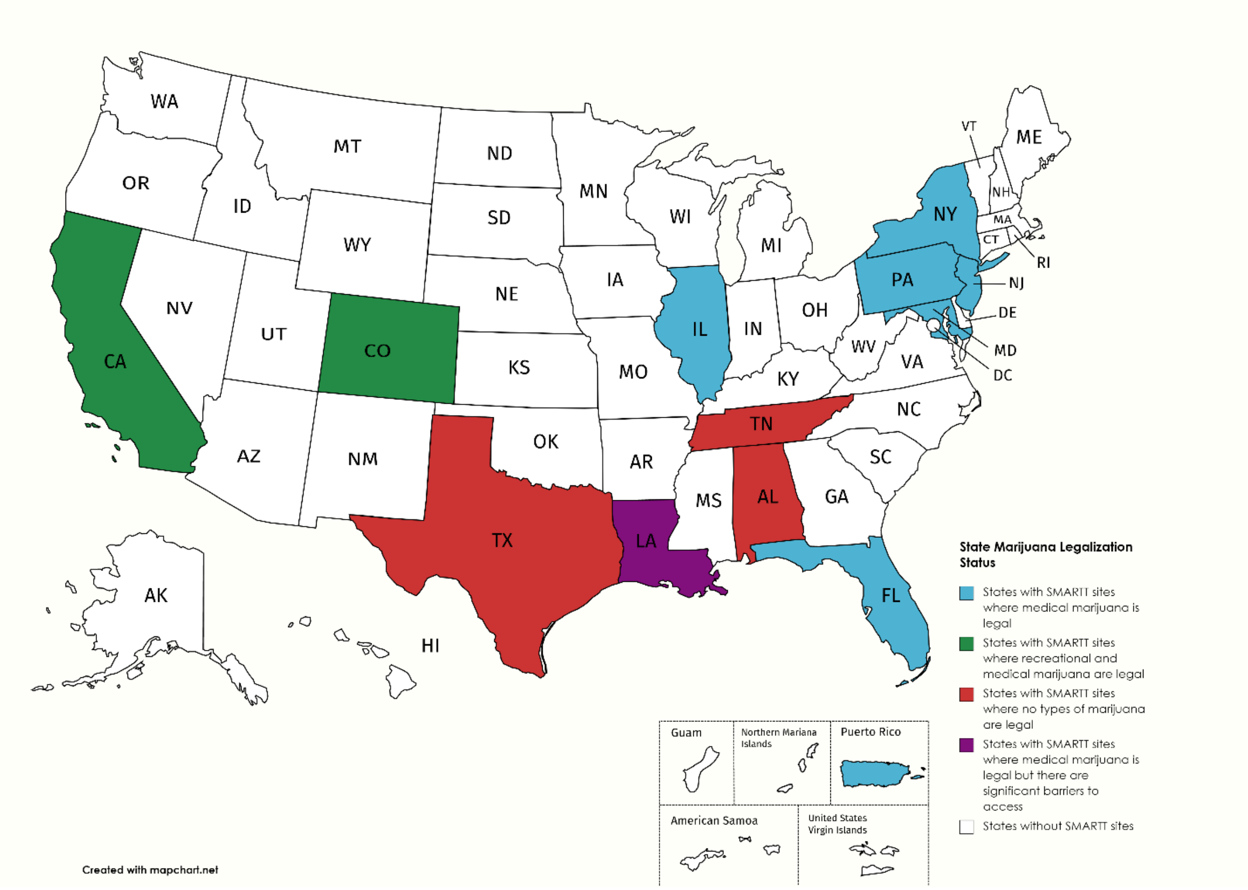

© 2021 Yee LM et al. *JAMA Network Open.*

eFigure 2. Study Population Derivation

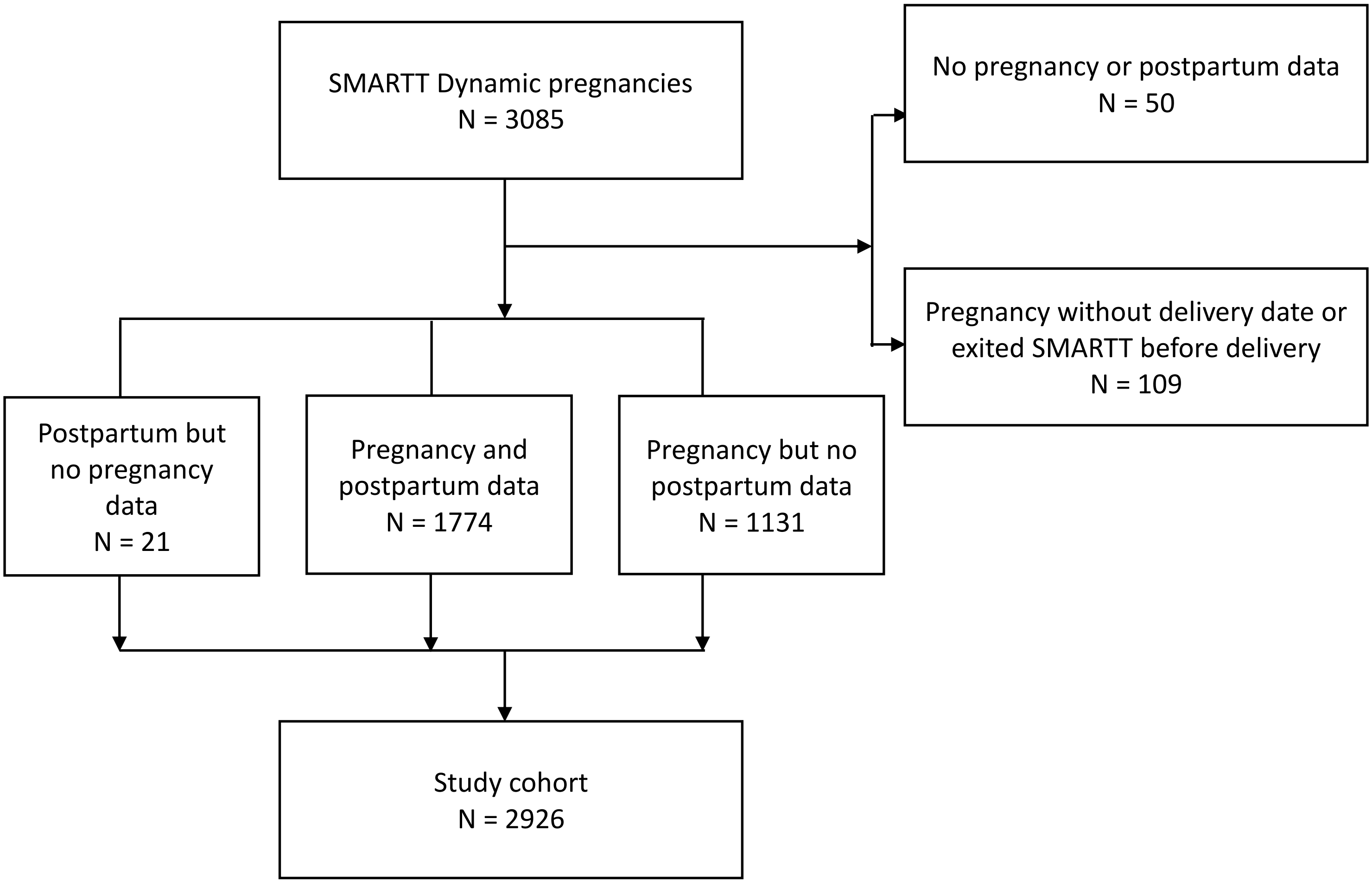

© 2021 Yee LM et al. *JAMA Network Open.*

eFigure 3. Prevalence and 95% Confidence Intervals of Substance Use During Pregnancy Among Persons Living With HIV by Delivery Year From 2007 to 2019

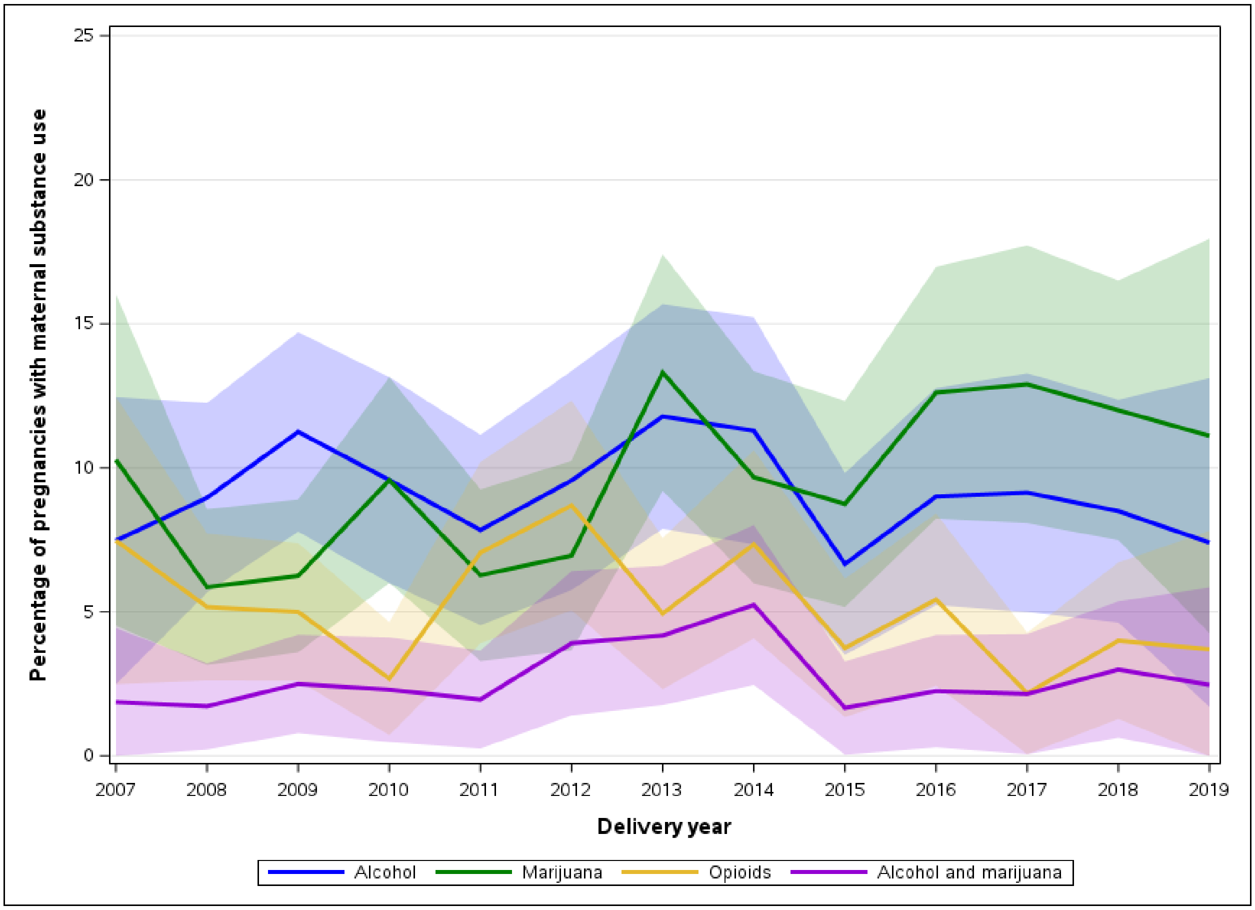

© 2021 Yee LM et al. *JAMA Network Open.*

eTable. Substance Use During Pregnancy and Postpartum by Year of Delivery Among Pregnant People Living With HIV From 2007 to 2019

Year of delivery	Substance use in pregnancy		Substance use postpartum	
	Proportion (%)	95% CI	Proportion (%)	95% CI
**ALCOHOL**				
2007-2008[1]	8.6	(5.81, 11.32)	36.2	(30.30, 42.14)
2009	11.3	(7.79, 14.71)	39.5	(32.34, 46.76)
2010	9.6	(6.01, 13.15)	44.3	(37.16, 51.49)
2011	7.8	(4.54, 11.14)	39.2	(32.19, 46.12)
2012	9.6	(5.76, 13.37)	53.8	(46.32, 61.37)
2013	11.8	(7.89, 15.69)	47.0	(39.47, 54.58)
2014	11.3	(7.35, 15.23)	52.1	(44.49, 59.75)
2015	6.7	(3.51, 9.82)	49.4	(41.94, 56.90)
2016	9.0	(5.24, 12.78)	46.6	(38.48, 54.67)
2017	9.1	(5.00, 13.28)	40.0	(31.40, 48.60)
2018-2019[1]	8.2	(4.98, 11.39)	42.1	(26.39, 57.82)
All Years	9.4	(8.31, 10.43)	44.4	(42.05, 46.66)
**MARIJUANA**				
2007-2008[1]	7.1	(4.53, 9.57)	10.2	(6.50, 13.97)
2009	6.3	(3.60, 8.90)	7.9	(3.91, 11.82)
2010	9.6	(6.01, 13.15)	10.8	(6.33, 15.29)
2011	6.3	(3.30, 9.25)	8.5	(4.49, 12.44)
2012	7.0	(3.67, 10.25)	11.8	(6.96, 16.71)
2013	13.3	(9.20, 17.42)	15.8	(10.32, 21.26)
2014	9.7	(6.00, 13.36)	17.6	(11.76, 23.39)
2015	8.8	(5.17, 12.33)	14.4	(9.15, 19.58)
2016	12.6	(8.24, 16.98)	19.2	(12.79, 25.57)
2017	12.9	(8.08, 17.72)	23.8	(16.37, 31.25)
2018-2019[1]	11.7	(7.98, 15.51)	23.7	(10.15, 37.21)
All Years	9.3	(8.24, 10.36)	13.6	(12.01, 15.18)
**OPIOIDS[2]**				
2007-2008[1]	5.8	(3.49, 8.09)		
2009	5.0	(2.61, 7.39)		
2010	2.7	(0.72, 4.64)		
2011	7.1	(3.91, 10.20)		

© 2021 Yee LM et al. *JAMA Network Open.*

2012	8.7	(5.05, 12.34)		
2013	4.9	(2.32, 7.56)		
2014	7.3	(4.08, 10.62)		
2015	3.8	(1.35, 6.15)		
2016	5.4	(2.44, 8.42)		
2017	2.2	(0.07, 4.28)		
2018-2019[1]	3.9	(1.65, 6.18)		
All Years	5.2	(4.40, 6.02)		
**CONCOMITANT ALCOHOL AND MARIJUANA**				
2007-2008[1]	1.8	(0.47, 3.06)	6.7	(3.62, 9.77)
2009	2.5	(0.79, 4.21)	7.3	(3.50, 11.19)
2010	2.3	(0.48, 4.12)	8.6	(4.59, 12.70)
2011	2.0	(0.26, 3.66)	4.2	(1.36, 7.11)
2012	3.9	(1.41, 6.42)	10.7	(6.00, 15.31)
2013	4.2	(1.76, 6.60)	11.3	(6.52, 16.10)
2014	5.2	(2.47, 8.02)	13.9	(8.65, 19.23)
2015	1.7	(0.05, 3.29)	10.5	(5.89, 15.04)
2016	2.3	(0.30, 4.21)	16.4	(10.42, 22.46)
2017	2.2	(0.06, 4.24)	12.8	(6.94, 18.66)
2018-2019[1]	2.8	(0.90, 4.79)	15.8	(4.18, 27.39)
All Years	2.8	(2.16, 3.35)	10.0	(8.57, 11.34)

HIV: Human Immunodeficiency Virus; CI: confidence interval

1. Years with partial data (2007 and 2019) grouped with nearest full year of data.
2. Opioid use not assessed postpartum.

© 2021 Yee LM et al. *JAMA Network Open.*

© 2021 Yee LM et al. *JAMA Network Open.*
